# Supplementary material for: Ptycho-endoscopy on a lensless ultrathin fiber bundle tip
Source: Light Sci Appl. 2024 Jul 17;13:168. doi: 10.1038/s41377-024-01510-5 (PMC11255264; doi:10.1038/s41377-024-01510-5)
Supplement: Supplementary file 1 — Supplementary Information [file 41377_2024_1510_MOESM1_ESM.pdf]

Supplementary Information for

Ptycho-endoscopy on a lensless ultrathin fiber bundle tip

Pengming Song[1,†,*], Ruihai Wang[1,†], Lars Loetgering[2], Jia Liu[1], Peter Vouras[3], Yujin Lee[1,4], Shaowei Jiang[1], Bin Feng[1], Andrew Maiden[5,6], Changhuei Yang[7], and Guoan Zheng[1,*]

[1]Department of Biomedical Engineering, University of Connecticut, Storrs, USA
[2]CarlZeiss AG, Carl Zeiss Promenade, Jena, Germany
[3]United States Department of Defense, Washington, D. C., USA
[4]School of Electrical and Electronic Engineering, Yonsei University, Seoul, Republic of Korea
[5]Department of Electronic and Electrical Engineering, University of Sheffield, Sheffield, UK
[6]Diamond Light Source, Harwell, Oxfordshire, UK
[7]Department of Electrical Engineering, California Institute of Technology, Pasadena, USA
[†]These authors contributed equally to this work
[*]Email: pengming.song@uconn.edu (P. S.), guoan.zheng@uconn.edu (G. Z.)

Contents

Supplementary Note 1: Blind recovery of endoscope's positional shifts in SAPE………….…………..….. 2-3
Supplementary Note 2: Defocusing of fiber bundle's proximal end for diffraction imaging..………………… 4-5
Supplementary Note 3: Recovery process of SAPE……………………………….………….….…………...6-7
Supplementary Note 4: Conventional ptychography and SAPE…………….……….……….…………….. 8-9
Supplementary Figures S5-S20……………..………..…………………...…………….………………………. 10-25
References …………..………..…………………...…………..…………..………….………………..…… 26

Supplementary Note 1: Blind recovery of endoscope's positional shifts in SAPE

In SAPE, the image acquisition process involves either manually holding or using a motorized stage to scan the distal end of a lensless endoscope tip. The endoscope tip can be a lensless fiber bundle or a lensless distal chip with coded surface. When operated with a lensless fiber bundle, the proximal end of the bundle remains stationary and is captured by a camera through a custom-built microscope system. A crucial aspect of SAPE's success is the accurate reconstruction of the object's exit wavefield $W(x, y)$, which hinges on determining the positional shifts (x_j, y_j) of the distal endoscope tip. When selecting the first captured image as the reference and comparing it to another captured image where the endoscope tip has shifted by (x_j, y_j), the cross-correlation map typically reveals two peaks. The primary peak at the origin (0, 0) corresponds to the stationary signal from the modulation profile of the fiber bundle or the coded surface. In contrast, the secondary peak at (x_j, y_j) indicates the positional shift of the object. To effectively infer the object shift information while reducing the influence of the stationary modulation profile, we perform the following processing for the captured images[1]: 1) We first estimate the probe's stationary modulation profile by averaging all captured images, 2) We then normalize each captured image against this mean profile to minimize the impact of the modulation profile:

$$I_{mean}(x, y) \;=\; \left(\sum_{j=1}^{J} I_j(x, y)\right)/J \tag{S1}$$

$$\hat{I}_j(x, y) = I_j(x, y)/I_{mean}(x, y) \tag{S2}$$

Here, $I_j(x, y)$ is the captured j^{th} measurement, J is the total number of acquisitions, and $\hat{I}_j(x, y)$ represents the processed image, wherein the effects of the stationary modulation profile are substantially reduced. With this processing, the initial positional shift of the object can be determined by identifying the peak of the cross-correlation between the reference image and the subsequent images:

$$(x_j, y_j) \;=\; \underset{(x_j, y_j)}{\operatorname{argmax}}\left(\hat{I}_{ref}(x, y) \star \hat{I}_j\left(x - x_j, y - y_j\right)\right) \tag{S3}$$

wherein the image $\hat{I}_1(x, y)$ is selected as the reference image $\hat{I}_{ref}(x, y)$, '$\star$' denotes the cross-correlation operation.

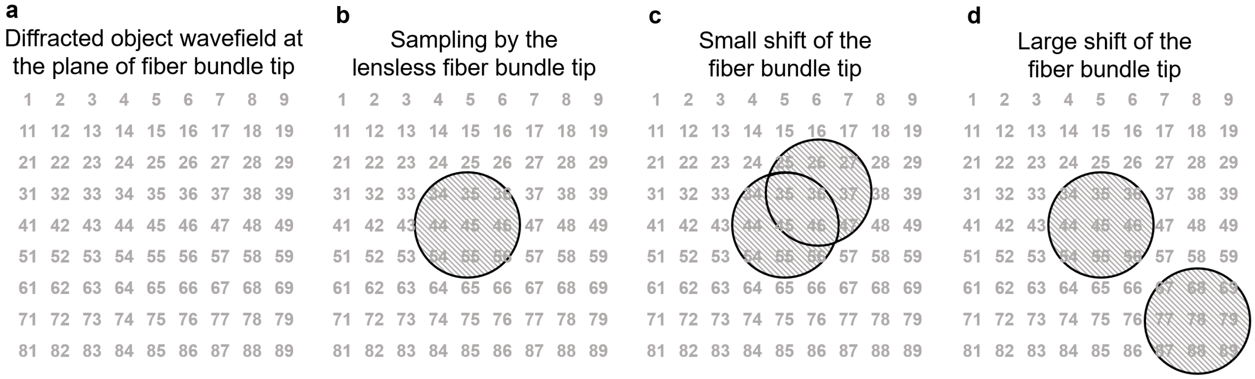

Fig. S1. **Positional tracking in SAPE**. (a) The conceptual representation of the diffracted wavefield at the distal endoscope tip. (b) Sampling area by the distal endoscope tip. (c) A small shift of the distal tip, highlighting the presence of an overlapping region suitable for cross-correlation analysis. (d) A large shift where the sampling areas do not or have little overlap, necessitating the use of a multi-reference strategy to perform cross-correlation analysis.

As the distal tip travels across different lateral positions, there may be instances where no or little overlap exists between the original reference image and the newly captured image. Figure S1a shows the conceptual representation of the diffracted wavefield at the plane of the distal endoscope tip. Figure S1b shows the sampling by the distal endoscope tip. Figures S1c and S1d differentiate between small and large positional shifts during acquisitions. The presence of an overlapping region in Fig. S1c allows for the inference of small shifts using the cross-correlation analysis previously described. In contrast, the large shift shown in Fig. S1d indicates that a single reference image is insufficient for accurate positional shift tracking due to a lack of overlap with the reference image. To address this challenge, we use multiple reference images in our methodology, selecting one reference image for every set

of 80 consecutive acquisitions. The other captured raw images are then processed relative to their nearest reference images.

Using Eq. (S3) in conjunction with the multi-reference strategy, we obtain initial estimations for the positional shifts of all measurements. However, these initial estimates often contain significant errors and are not optimal. To enhance accuracy, we employ a refinement step by shifting back the raw images based on the estimated shifts. This technique involves generating an updated reference image with the following equation[1]:

$$I_{ref}^{update}(x,y) = \sum_{j=1}^{J} I_j\left(x + x_j, y + y_j\right) \quad \text{(S4)}$$

Subsequently, the positional shifts are refined as:

$$\left(x_j^{update}, y_j^{update}\right) = \underset{(x_j, y_j)}{\text{argmax}} \left(I_{ref}^{update}(x,y) \star \hat{I}_j\left(x - x_j, y - y_j\right)\right) \quad \text{(S5)}$$

This refinement process is typically iterated twice to achieve the final, more accurate positional shifts. Regarding the computational time for tracking the 2D positional shifts, we typically process a set of 500 measurements, each with a dimension of 1024×1024 pixels. The processing time for this tracking operation is approximately 7 seconds in our experiments.

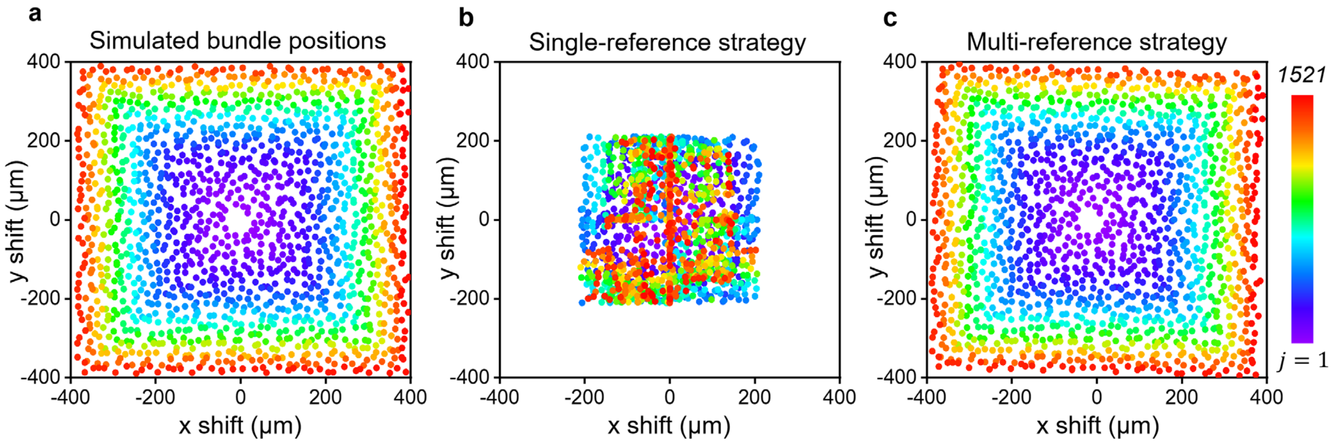

Fig. S2. Validation of the positional tracking process of SAPE. (a) Simulated ground-truth positions for the distal endoscope tip. (b) Estimated positions using a single reference image. (c) Estimated positions using the multi-reference strategy with the refinement step.

To demonstrate the effectiveness of our positional tracking process of SAPE, we performed a simulation study in Fig. S2. The simulated ground-truth positions are presented in Fig. S2a. When relying on a single reference image for positional estimation, we observed that the accuracy of recovery is largely limited to areas immediately surrounding the reference point, as depicted in Fig. S2b. Conversely, implementing a multi-reference strategy addresses this issue, as shown in Fig. S2c. The mean error between the ground-truth positions and our estimations is 0.12 μm in this simulation study, underscoring the efficacy of our positional tracking approach.

Supplementary Note 2: Defocusing of the fiber bundle's proximal end for diffraction imaging

In SAPE, the lensless fiber bundle's distal end is shifted across various lateral positions for data acquisition. At the same time, its proximal end is imaged by a custom-built microscope. A consideration in this process involves the intentional defocusing of the proximal end by a small distance h. This defocus distance allows for the conversion of phase information from the object's diffracted wavefield into discernible intensity variations on the camera sensor, which is essential for the accurate detection and reconstruction of the final image. Assuming the fiber bundle profile $FB(x,y)$ remains unchanged during the acquisition process, we consider a simplified forward imaging model of SAPE as follows:

$$I_j(x,y) = \left|\{W(x,y) \cdot FB(x-x_j, y-y_j)\} * psf_{free}(h)\right|^2 \quad \text{(S6)}$$

where $I_j(x,y)$ denotes the jth acquired diffraction pattern corresponding to the distal fiber bundle shift (x_j, y_j), and $W(x,y)$ denotes the object's diffracted wavefield we aim to recover. The convolution kernel $psf_{free}(h)$ models free-space light propagation over a distance h at the proximal end of the fiber bundle. When h = 0, Eq. (S6) simplifies to:

$$I_j(x,y) = \left|W(x,y) \cdot FB(x-x_j, y-y_j)\right|^2 = |W(x,y)|^2 \cdot \left|FB(x-x_j, y-y_j)\right|^2 \quad \text{(S7)}$$

In this scenario, only the amplitude information is captured, and the phase information is irretrievably lost. Similar effects have also been demonstrated and validated in near-field Fourier ptychography[2].

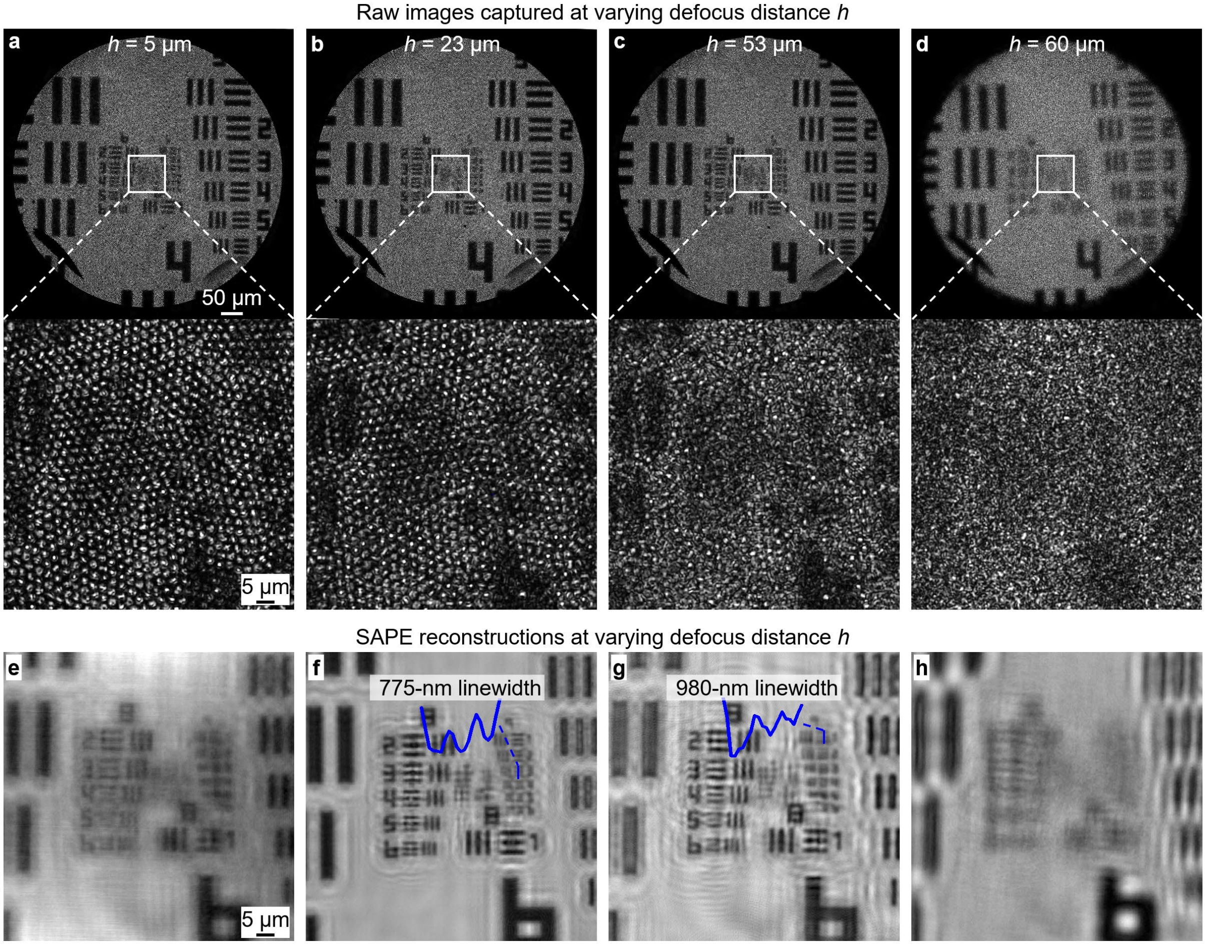

Fig. S3. Impact of proximal fiber bundle's defocus distance on SAPE imaging. (a-d) The raw images of a resolution target captured with defocus distances of 5 µm, 23 µm, 53 µm, and 60 µm. A larger defocus distance facilitates the conversion of phase information into intensity variations for detection. However, a larger defocus distance also leads to difficulty for accurate positional tracking. (e-h) The SAPE reconstructions corresponding to different defocus distances, revealing the balance necessary for optimal image reconstruction.

Conversely, if h is an infinitely large value, Eq. (S6) becomes:

$$I_j(x,y) = \left|FT\{W(x,y) \cdot FB(x - x_j, y - y_j)\}\right|^2 \tag{S8}$$

where 'FT' denotes the Fourier transform indicative of far-field propagation. Eq. (S8) describes an imaging model similar to that of conventional ptychography[3]. As a coherent diffraction imaging modality, light 'diffraction' is necessary for converting the phase information into intensity variations with either Eq. (S6) or (S8). We also note that, when implementing SAPE with distal-chip endoscopy, we replace the small lens of the miniaturized camera with a coded surface directly attached on top of the image sensor's coverglass. The defocusing distance h in Eq. (S6) indicates the free space between the coded surface and the pixel array of the image sensor.

Our experiments have shown that an excessively small defocus distance ineffectively translates phase into intensity, thus degrading the SAPE reconstruction. On the other hand, a large defocus distance introduces complications in recovering positional shifts due to pronounced diffraction effects. To balance these considerations, we selected a defocus distance of approximately 20 μm for our implementation. Figure S3 illustrates the significance of selecting an appropriate defocus distance. Particularly, larger defocus distances reduce the contrast in raw images, as seen in Figure S3d, complicating the blind recovery of positional shifts. This can lead to inaccurately estimated shifts, which noticeably degrade the resolution of the SAPE reconstruction in Fig. S3h. Similarly, a small defocus distance, shown in Fig. S3a, adversely affects SAPE quality in Fig. S3e since phase information is not adequately converted into intensity variations.

Supplementary Note 3: Recovery process of SAPE

We develop the following reconstruction procedures with spatiotemporal decomposition of the modulation profile of the endoscope probe. In our implementation, the captured images $I_j(x,y)$ (j = 1,2, ... J) have an image dimension of $m \times n$ pixels, the wavefront $W(x,y)$ have an image dimension of $M \times N$ pixels. Here m, n, M, N are even numbers and $M > m$, $N > n$.

Step 1: The object's diffracted wavefield $W(x,y)$ at the distal tip is initialized by stitching together the captured raw images, using the estimated positional shifts from Supplementary Note 1:

$$I_j^{shift}(x,y) = I_j(x-(x_j-\lfloor x_j \rfloor), y-(y_j-\lfloor y_j \rfloor) \quad \text{(S9)}$$

$$W\left(x+\frac{M-m}{2}+\lfloor x_j \rfloor, y+\frac{N-n}{2}+\lfloor y_j \rfloor\right) = \sqrt{I_j^{shift}(x,y)}, \ (x=1,2,\dots m; y=1,2,\dots n) \quad \text{(S10)}$$

Here, $I_j(x,y)$ is the jth captured image, (x_j, y_j) is the positional shift of the jth measurement, '$\lfloor \cdot \rfloor$' denotes the floor function. The position-dependent modulation profiles $FB_{sj}(x,y)$ (s = 1,2, ... S) (j = 1,2, ... J) are initialized as:

$$FB_{sj}(x,y) = rand(m,n) \cdot \sum_j \sqrt{I_j(x,y)}/J, \quad \text{(S11)}$$

where the subscript 's' denotes different spatially incoherent modes, $rand(m,n)$ denotes a random matrix with the dimensional of $m \times n$ pixels.

Step 2: We crop a sub-region from the wavefield as:

$$W_j(x,y) = W(x+\frac{M-m}{2}+\lfloor x_j \rfloor, y+\frac{N-n}{2}+\lfloor y_j \rfloor), \ (x=1,2,\dots m; y=1,2,\dots n) \quad \text{(S12)}$$

We then shift the sth spatially incoherent mode of the modulation profile, $FB_s(x,y)$, by $(x_j-\lfloor x_j \rfloor, \ y_j-\lfloor y_j \rfloor)$ via

$$FB_{sj}^{shift}(x,y) = FB_{sj}(x+(x_j-\lfloor x_j \rfloor), y+(y_j-\lfloor y_j \rfloor)) \quad \text{(S13)}$$

Step 3: The exit wave $\varphi_{sj}(x,y)$ is given by the product of $FB_{sj}^{shift}(x,y)$ and the sub-region of the wavefront:

$$\varphi_{sj}(x,y) = W_j(x,y) \cdot FB_{sj}^{shift}(x,y) \quad \text{(S14)}$$

where the symbol '·' indicates point-wise multiplication.

Step 4: We propagate the exit wave $\varphi_{sj}(x,y)$ to the sensor plane:

$$\phi_{sj}(x,y) = \varphi_{sj}(x,y) * psf_h(x,y) \quad \text{(S15)}$$

where $psf_h(x,y)$ denotes the free-space propagation of a distance h, the symbol '*' denotes the convolution operation.

Step 5: We repeat steps 2-4 for all spatially incoherent modes $FB_{sj}(x,y)$ $(s = 1,2, \dots)$. The estimated image at the sensor plane is essentially a composite of various spatially incoherent modes, as indicated by the subscript 's' in the summation symbol as follows:

$$I_j^{est}(x,y) = \sum_s \left|\phi_{sj}(x,y)\right|^2 \quad \text{(S16)}$$

Step 6: The wavefront $\phi_{sj}(x,y)$ at the sensor plane is updated as follows:

$$\phi_{sj}^{update}(x,y) = \phi_{sj}(x,y) \cdot \frac{\sqrt{I_j(x,y)}}{\sqrt{I_j^{est}(x,y)}} \quad \text{(S17)}$$

The exit wave $\varphi_{sj}(x,y)$ is then updated by back-propagating $\phi_{sj}^{update}(x,y)$ of a distance h:

$$\varphi_{sj}^{update}(x,y) = \phi_{sj}^{update}(x,y) * psf_{-h}(x,y) \quad \text{(S18)}$$

Step 7: With the updated exit wave $\varphi_{sj}^{update}(x,y)$, we update the corresponding sub-region of $W(x,y)$ and the modulation profile using the rPIE algorithm[4]:

$$W_j^{update}(x,y) = W_j(x,y) + \frac{conj\left(FB_{sj}^{shift}(x,y)\right) \cdot (\varphi_{sj}^{update}(x,y) - \varphi_{sj}(x,y))}{\left|FB_{sj}^{shift}(x,y)\right|_{max}^2} \quad \text{(S19)}$$

$$FB_{sj}^{shift,update}(x,y) = FB_{sj}^{shift}(x,y) + \frac{conj\left(W_j(x,y)\right)\cdot(\varphi_{sj}^{update}(x,y) - \varphi_{sj}(x,y))}{|W_j(x,y)|_{max}^2} \tag{S20}$$

Step 8: The wavefront $W(x,y)$ is obtained from the updated wavefront $W_j^{update}(x,y)$ as follows:

$$W\left(x + \frac{M-m}{2} + \lfloor x_j \rfloor, y + \frac{N-n}{2} + \lfloor y_j \rfloor\right) = W_j^{update}(x,y) \tag{S21}$$

The sth spatially incoherent mode is obtained from the updated shifted profile $FB_{sj}^{shift,update}(x,y)$ as follows:

$$FB_{sj}(x,y) = FB_{sj}^{shift,update}\left(x - \left(x_j - \lfloor x_j \rfloor\right), y - \left(y_j - \lfloor y_j \rfloor\right)\right) \tag{S22}$$

We repeat steps 6-8 for all incoherent modes of $FB_{sj}(x,y)$ $(s = 1,2,3, \ldots)$.

Step 9: Steps 2-8 are repeated for all the captured images to complete one iteration. The reconstructed position-dependent modulation profiles $FB_{sj}(x,y)$ (j $= 1,2, \ldots J$) are linked together by a projecting them into a lower T-dimensional space using singular value decomposition (SVD), with T << J.

$$\left[P_{s[.]}, D_s, V_s\right] = truncated\_SVD(FB_{s[.]}, T) \tag{S23}$$

where $FB_{s[.]}$ is formed by converting each 2D profile of $FB_{sj}(x,y)$ into a vector and then concatenating all J vectors column-wise. $P_{s[.]}$ is the projected orthogonal matrix with T columns, and each column can be reshaped into orthogonal mode $P_{st}(x,y)$, with $t = 1,2, \ldots T$. D_s is a T-by-T diagonal matrix of singular values, and V_s is a J-by-T complex conjugated orthonormal evolution matrix. The position-dependent modulation profile $FB_{sj}(x,y)$ is updated by its lower dimensional representation as follows:

$$FB_{sj}(x,y) = \sum_t (P_{st}(x,y) \cdot \alpha_{tj}) \tag{S24}$$

where $\alpha_{tj} = (D_s \times V_s^H)_{tj}$, representing the element in the tth row and the jth column of the product between D_s and the Hermitian transpose of V_s.

Step 10: Steps 2-9 are repeated until a convergence condition is fulfilled -- either a fixed number of iterations or stagnation of an error metric.

In the current implementation, we did not optimize the reconstruction speed using Compute Unified Device Architecture (CUDA). For 2D reconstruction using 500 measurements, the reconstruction takes 5-10 minutes depending on the number of iterations. Once the 2D reconstruction is completed, we can propagate it to different axial positions. It typically takes 1~2 minutes to obtain the topographic map. Real-time processing is not feasible in the current implementation. However, the rapid development and increasing power of recent graphical processing units (GPUs), driven by the demands of deep learning, offer promising opportunities for accelerating the reconstruction process. By leveraging the parallel processing capabilities of modern GPUs and optimizing the algorithms using CUDA or other GPU-accelerated libraries, we expect to significantly reduce the computation time. This could potentially bring the reconstruction speed closer to video rate in future implementations.

For additional details on the reconstruction routines and related techniques, we direct the interested readers to the following related resources[2, 4, 5, 6]. The data of the captured raw images and the corresponding SAPE constructions of the resolution target in the main text of Figs. 2d, 3d, 5d can be accessed in Ref.[7].

Supplementary Note 4: Conventional ptychography and SAPE

SAPE advances the principle of conventional ptychography by utilizing a spatially confined fiber bundle profile as a coded surface to modulate the object's diffracted wavefield. This method also departs from both conventional ptychography[3] and near-field ptychography[8] by synthesizing information at the fiber bundle's diffraction plane rather than at the object plane. Figure S4 illustrates the distinctions between conventional ptychography and SAPE, emphasizing their respective approaches to image capture and reconstruction. In both methods, the object is placed within the spatial domain, and detection occurs at the diffraction plane. Conventional ptychography utilizes a spatially-confined probe beam, $P(x, y)$, for object illumination. This probe beam interacts with the object translated to different lateral positions, $O(x - x_i, y - y_i)$, and the resultant diffracted wavefield travels a distance h to reach the detector. During the reconstruction process of conventional ptychography, the diffraction patterns are used to recover the corresponding spatially-confined regions in real space, thereby broadening the imaging field as depicted in Fig. S4a. The resolution achieved through this method is determined by the detector's spanning angle. An important aspect of this process is the overlap between successive translated positions of the object, which ensures ample data redundancy essential for successful phase retrieval.

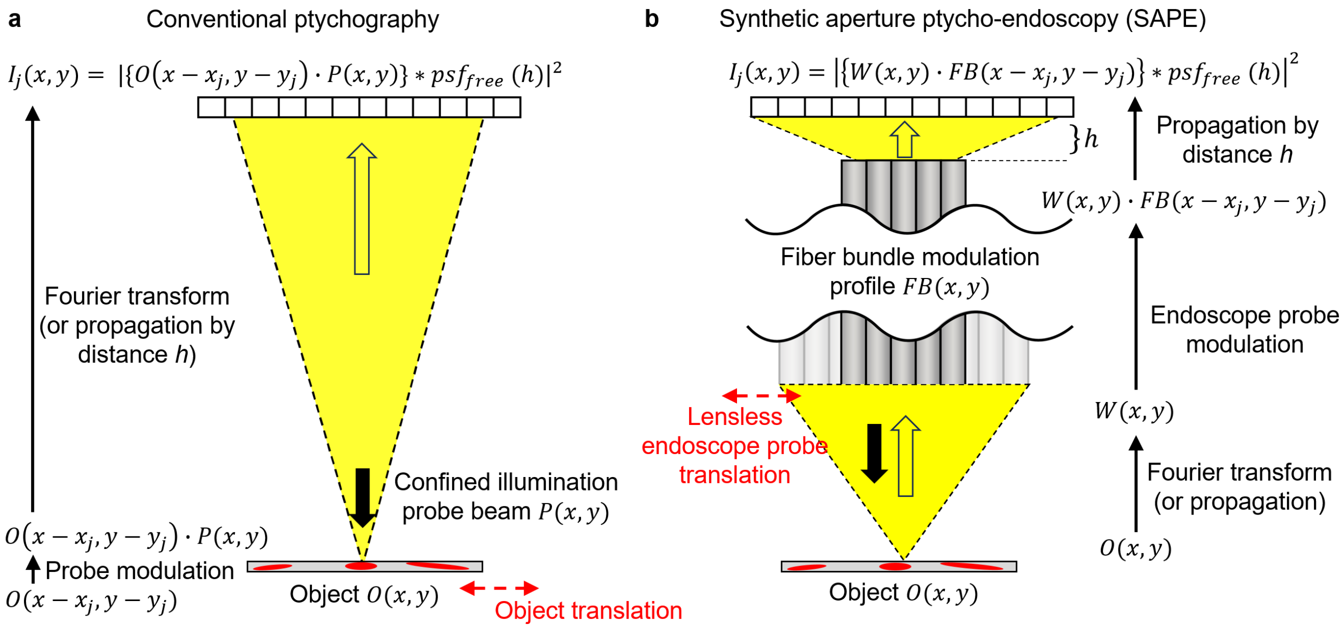

Fig. S4. Comparison between conventional ptychography and SAPE. (a) Conventional ptychography translates the object over a confined probe beam in the spatial domain and acquires the diffraction data at the far-field. The light diffraction effect in conventional ptychography refers to the propagation of light between the object and the detector. During the reconstruction process, conventional ptychography stitches the information in the spatial domain to expand the field of view of the object. (b) SAPE illuminates the object with an extended wave and translates the distal fiber bundle tip for data acquisition. The illumination can be an external collimated beam or light emitting from a single-mode fiber core. The fiber bundle could cause partial obstruction of the illumination beam when the specimen is placed too close to the fiber bundle tip. The light diffraction effect in SAPE refers to the propagation of light between the proximal fiber bundle tip and the detector. In addition to the fiber bundle implementation, SAPE can also be adapted for distal-chip endoscopes, where a miniaturized camera is directly integrated at the distal end of the imaging probe. In this configuration, the small lens element of the distal camera can be replaced with a coded surface, such as a flexible coded thin film, a diffuser or, a disorder-engineered mask. The coded surface modulates the object's diffracted wavefield in a similar manner to the fiber bundle tip. During the data acquisition process, the endoscope probe with the coded surface is translated to different positions, and the modulated intensity patterns are captured by the distal camera. In the reconstruction process, SAPE stitches the information at the plane of distal tip, instead of at the object plane. SAPE can widen the field of view in real space and expand the spatial-frequency bandwidth in reciprocal space at the same time.

In SAPE, we illuminate the entire object with an extended beam. In the reflective model shown in Fig. S4b, a spherical wave from a single mode fiber or a collimated beam can be used for sample illumination. The diffracted

wavefield from the object then propagates for a distance *d* and reaches the plane of the distal fiber bundle tip. The spatially-confined circular profile of the fiber bundle modulates the object's diffracted wavefield in a point-wise multiplication process, similar to the object-probe interaction in conventional ptychography. In this regard, the fiber bundle facet in SAPE serves as the equivalent spatially-confined probe beam as that in conventional ptychography. The modulated wavefield then propagates for a small distance *h* to the detector plane. Alternatively, SAPE can be implemented in distal-chip endoscopy where a miniaturized camera is placed at the distal end of the imaging probe. In this setting, the small lens element of the miniaturized camera can be replaced by a thin coded layer on the sensor. In the reconstruction process, SAPE iteratively stitches the information at the plane of the distal fiber bundle tip. By back-propagating this wavefield back to the object plane, SAPE can simultaneously widen the imaging field of view in real space and expand the spatial-frequency bandwidth in reciprocal space. If the distance between the object and the distal fiber bundle tip is an infinitely large value, SAPE can be viewed as Fourier ptychography[9], where the information is stitched and recovered at the reciprocal space.

The unique properties of SAPE can be summarized as follows: First, SAPE uniquely recovers the object's diffracted wavefield at the diffraction plane, which allows for both an expanded imaging field of view and an increased spatial-frequency bandwidth. Second, the modulation profile of the distal fiber bundle tip or the coded surface on a distal-chip, once characterized in a calibration experiment, serves as a good initial guess for ptychographic phase retrieval, often facilitating higher quality reconstruction than is typically achieved with conventional blind ptychography, where both the object and the illumination probe needed to be jointly recovered[10, 11]. Third, unlike conventional ptychography, which necessitates a point-wise multiplication model valid only for thin samples, SAPE reconstructs the wavefield at the plane of the lensless endoscope tip, thereby eliminating the need for modelling the interaction between the object and the illumination beam[12]. This approach renders the object's thickness irrelevant to the reconstruction process, allowing for the digital propagation of the recovered wavefront to any axial position for post-measurement refocusing[13]. Fourth, SAPE can be readily adapted for industrial endoscopes with a miniaturized camera at the distal end. This adaptation of SAPE for industrial endoscopes enables high-resolution imaging and 3D topographic reconstruction in a compact, lens-free design, making it particularly suitable for non-destructive testing and quality control applications.

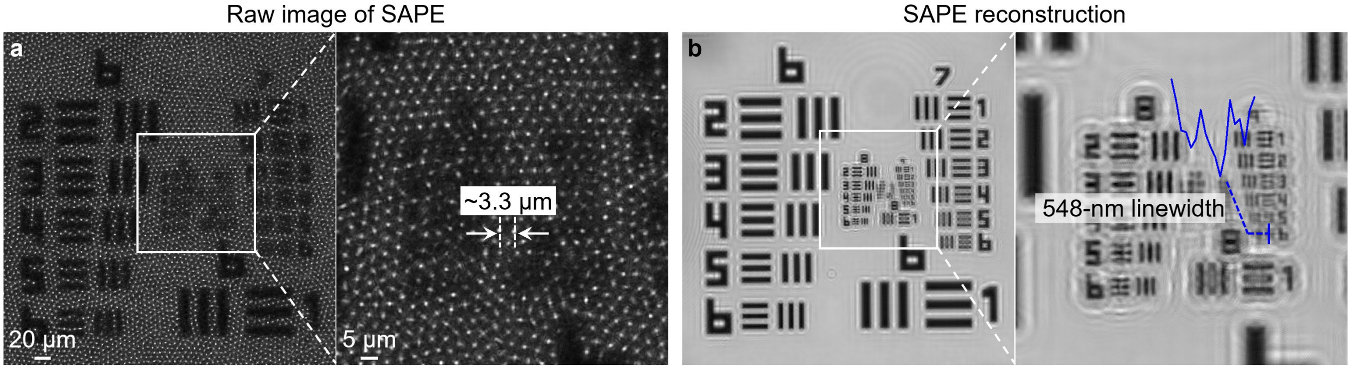

Fig. S5. Imaging performance of SAPE characterized using a resolution target. (a) A captured raw image of a resolution target by mounting the distal fiber bundle tip on a motorized stage and illuminated with a 405-nm laser diode. (b) The image reconstructed by SAPE, which successfully resolves a linewidth of 548 nm within group 9, element 6, representing one of the highest resolutions attained using a fiber bundle tip.

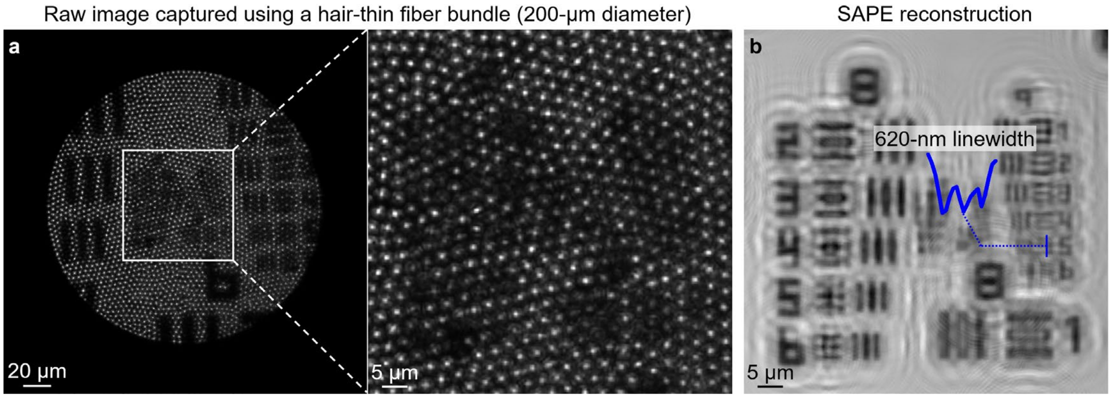

Fig. S6. SAPE imaging performance with a 200-μm diameter hair-thin fiber bundle (Model: FIGH-03-200S). (a) The captured raw image with a 405 nm laser diode and its zoomed-in view. (b) The SAPE reconstruction, where we can resolve a 620-nm linewidth of group 9, element 5 on the resolution target.

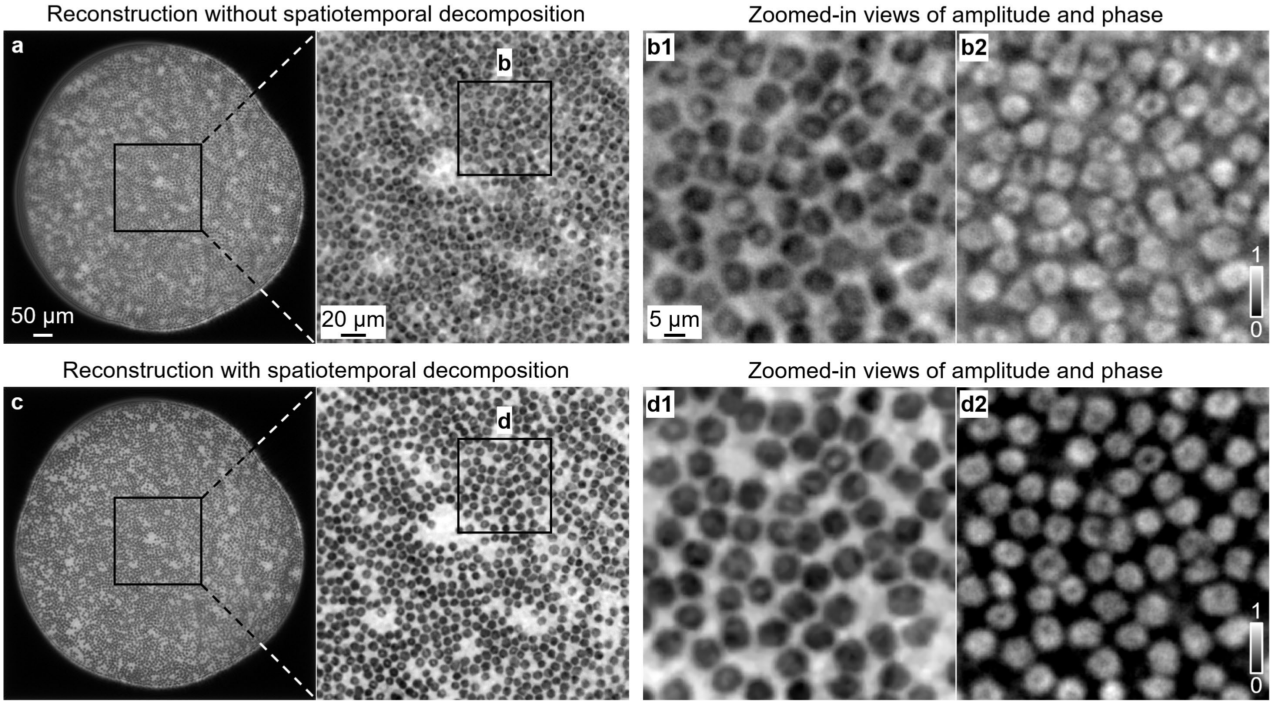

Fig. S7. Comparative SAPE reconstructions of a blood smear slide. (a) SAPE reconstruction without spatiotemporal decomposition, applying a consistent modulation profile for the fiber bundle at all translated positions. (b) A magnified view of the region marked in (a), showing the amplitude (b1) and phase (b2) of the blood cells. (c) SAPE reconstruction with spatiotemporal decomposition implemented. (d) A magnified view of the region marked in (c), showing the amplitude (d1) and phase (d2) with better image quality.

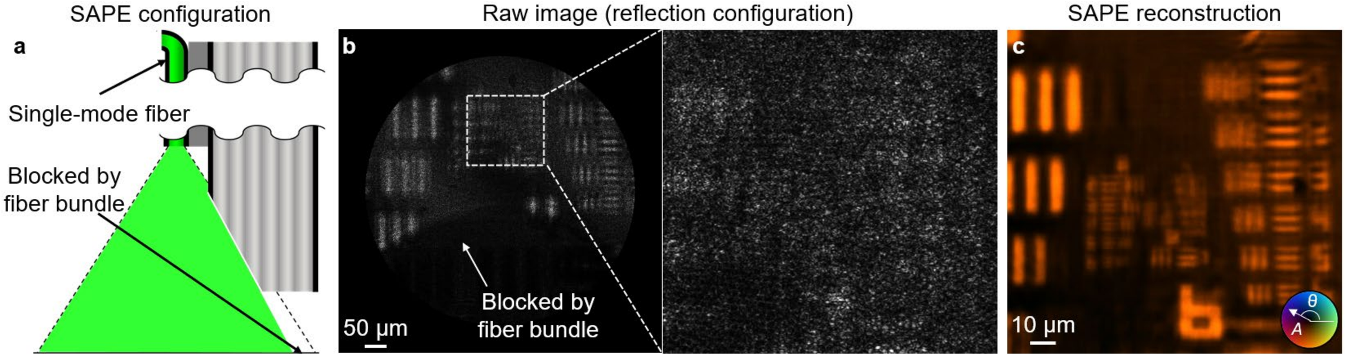

Fig. S8. Reflective configuration of SAPE. (a) In the reflection setup of SAPE, an external collimated beam or a separate single-mode fiber can be used to deliver laser light for object illumination. The fiber bundle could cause partial obstruction of the illumination beam when the specimen is placed close to the fiber bundle tip. An alternative implementation for distal-chip SAPE is to employ a small beamsplitter on top of a coded sensor and deliver collimated laser beam to the specimen. (b) The captured raw image of a resolution target, with the bottom region partially obscured by the distal fiber bundle tip. (c) The SAPE recovery of the zoomed-in area from (b), demonstrating the technique's effectiveness despite the partial obstruction.

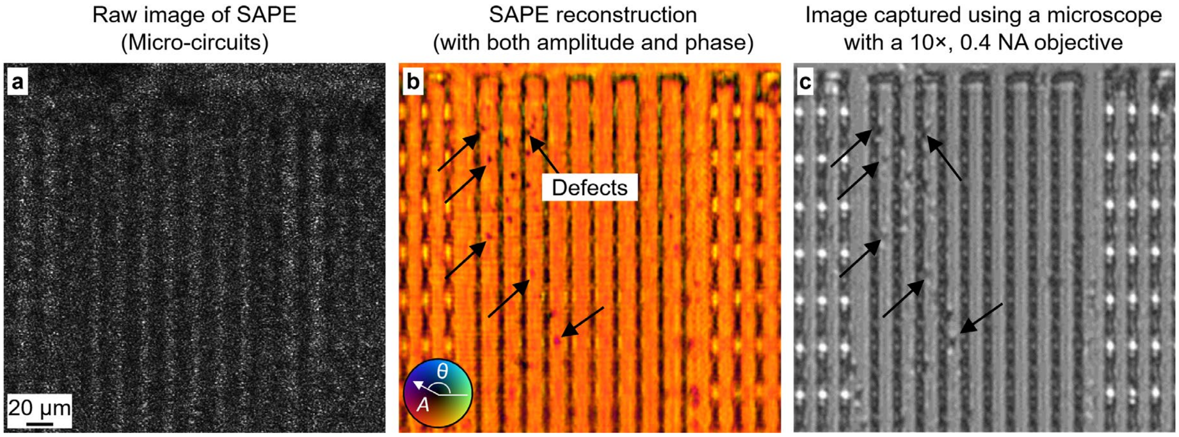

Fig. S9. Micro-circuit imaging via SAPE and conventional lens-based microscopy. (a) The captured raw image of micro-circuits using the lensless ultrathin fiber bundle tip. (b) The SAPE reconstruction with amplitude represented by grey values while phase by color hues. (c) Image of the same region captured using a regular bright-field microscope with a 10×, 0.4 NA Nikon objective lens. Both SAPE and the traditional microscopy method effectively identify defects in the micro-circuits, demonstrating the efficacy of SAPE in detailed imaging comparable to standard lens-based microscopic techniques.

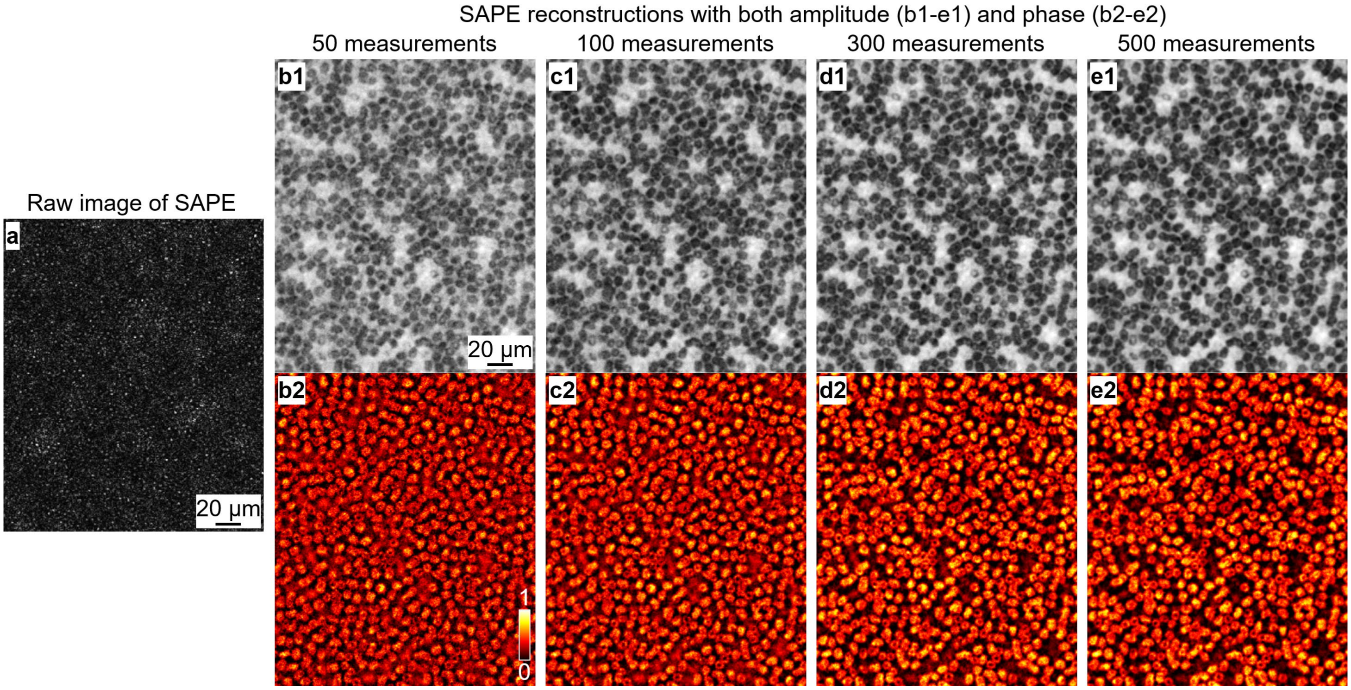

Fig. S10. SAPE reconstructions of a blood smear sample. (a)The captured raw image using a transmission configuration. The SAPE reconstructions using 50 images (a), 100 images (b), 300 images (c), and 500 images (d).

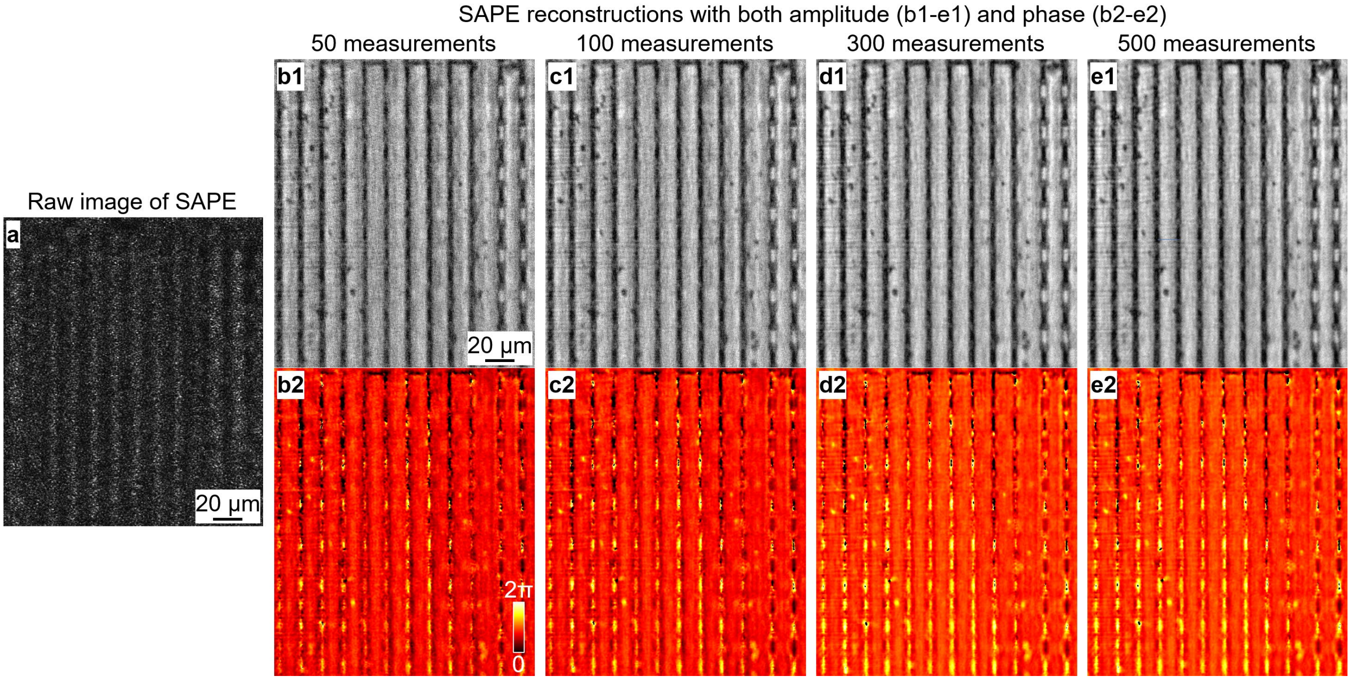

Fig. S11. SAPE reconstructions of micro-circuits. (a) The captured raw image using a reflective configuration. The SAPE reconstructions using 50 images (a), 100 images (b), 300 images (c), and 500 images (d).

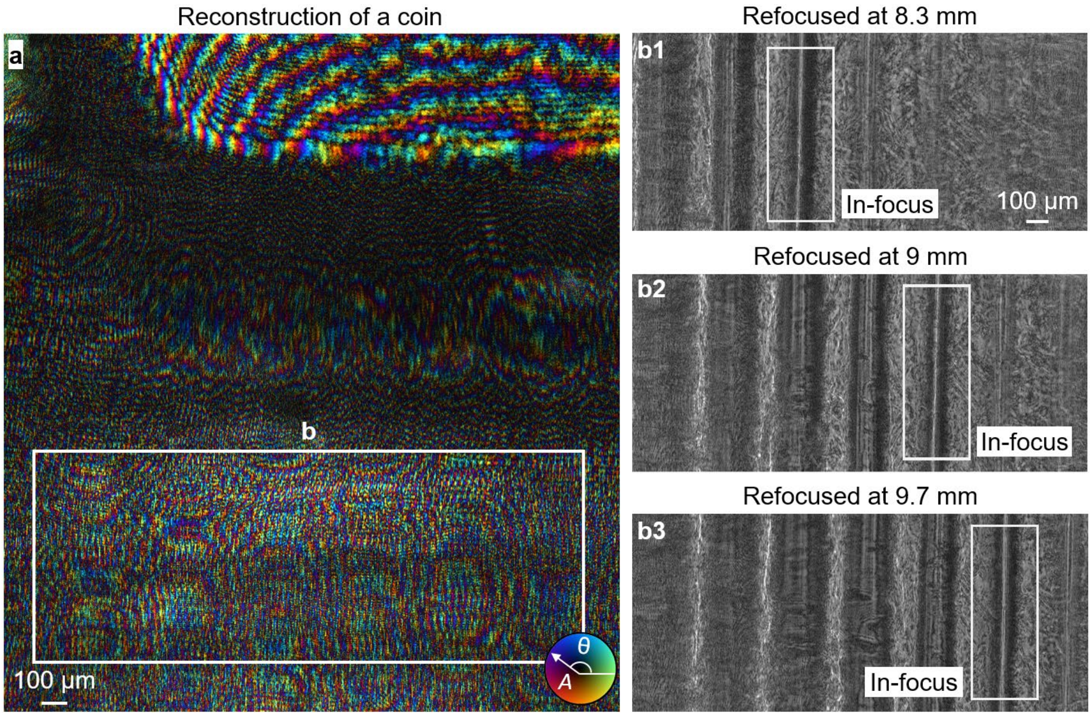

Fig. S12. Proof-of-concept demonstration of the SAPE's digital refocusing capability for 3D topographic imaging. A motorized stage is used to translate a lensless distal-chip SAPE probe across a coin sample for image acquisition. (a) The recovered complex wavefield at the distal facet of the lensless endoscope, with amplitude represented by grey values while phase by colour hues. (b1-b3) The refocused wavefields at z = 8.3 mm, 9 mm, and 9.7 mm toward the object, each highlighting different in-focus region of the coin. The 3D height map of the coin object can then be derived by selecting regions with maximized contrast from the digitally refocused images[14]. By adopting the SAPE concept in industrial distal-chip endoscopes, we can achieve high-resolution object inspection, enabling the visualization of intricate surface details and topographic variations that are crucial for quality control and failure analysis.

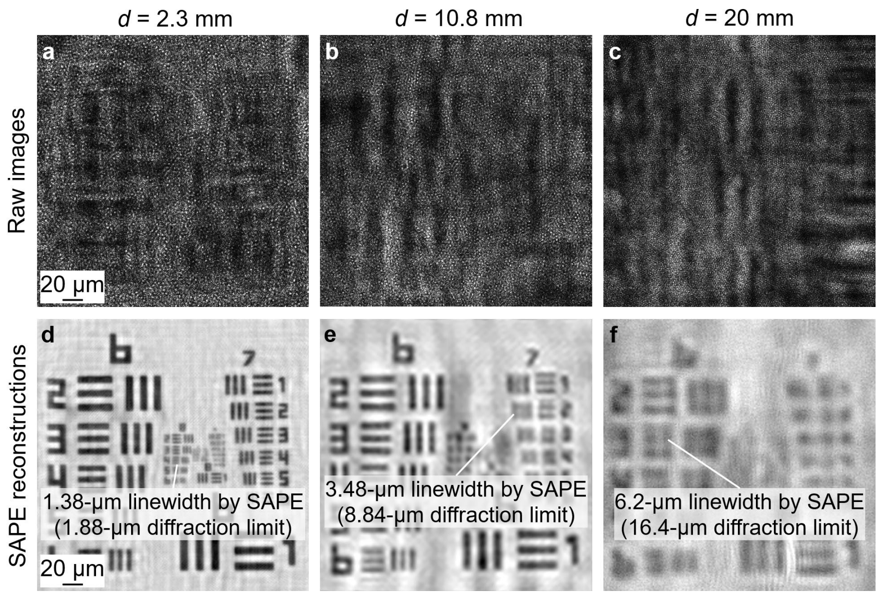

Fig. S13. Experimental validation of the super-resolution imaging capability with an extended depth of field. The captured raw images of a resolution target positioned at distances of 2.3 mm (a), 10.8 mm (b), and 20 mm (c) from the distal fiber bundle tip. The diameter of the employed fiber bundle tip is 650 μm in these experiments. (d-f) The SAPE reconstructions corresponding to different object-to-tip distances. The achieved resolutions surpass the corresponding diffraction limits determined by the maximum collection angle of the fiber bundle.

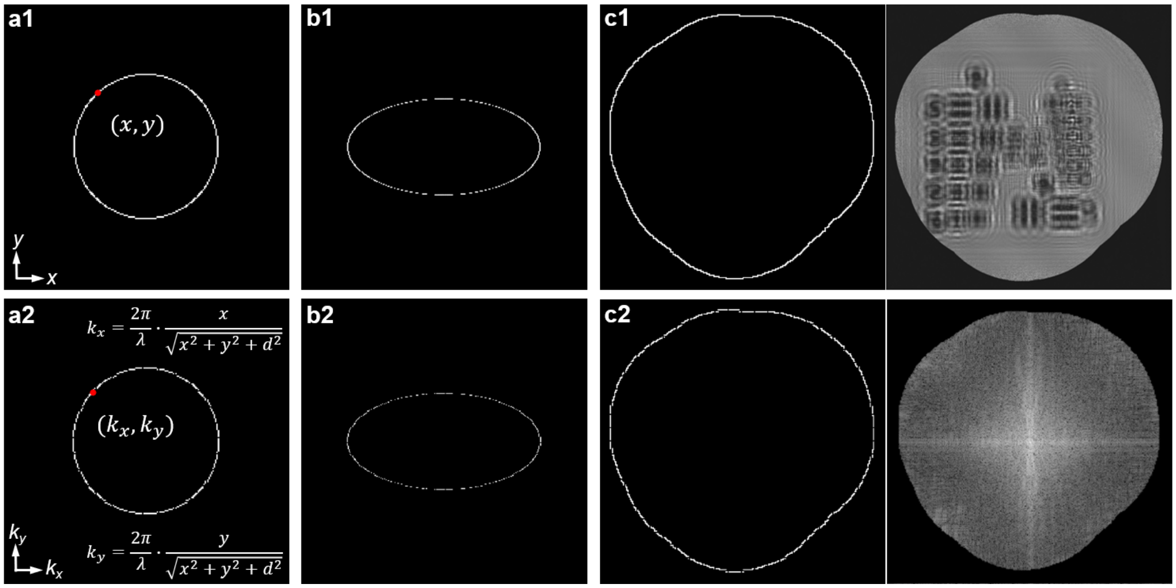

Fig. S14. Comparative areas in the spatial and Fourier domains. (a1) A circle in the spatial domain and (a2) its corresponding coverage in the Fourier domain. (b1) An oval shape in the spatial domain and (b2) its counterpart in the Fourier domain. (c1-c2) The outlined coverages are related to Figures 5(b) and 5(c) of the main manuscript.

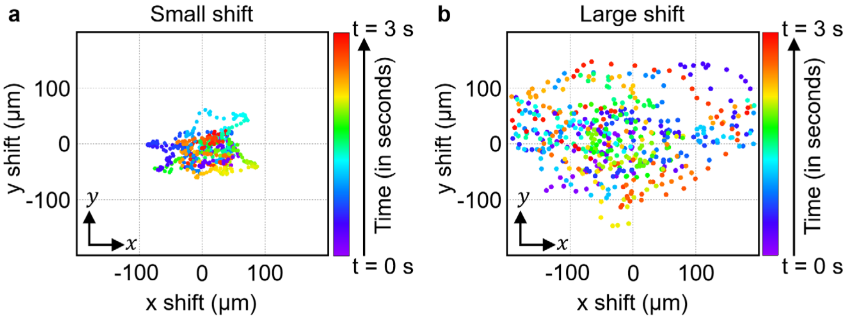

Fig. S15. Recovered handheld trajectories of the distal fiber bundle tip. With adequate training, an operator can effectively control the range of hand motion to optimize the experiment's outcome. (a) Small shifts with an average 5.33-µm step size. (b) Relatively large shifts with an average 27.1-µm step size.

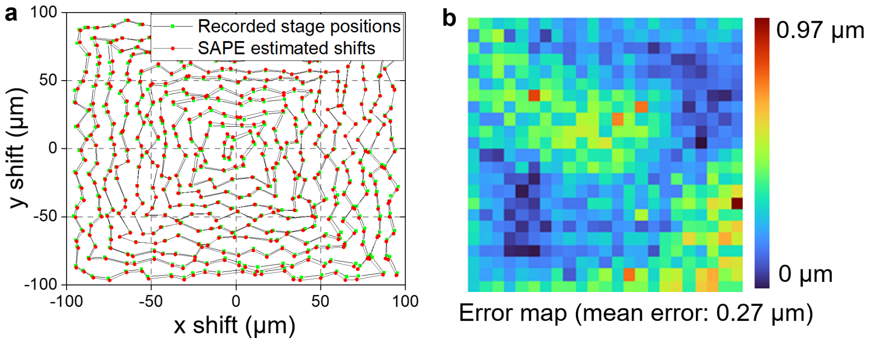

Fig. S16. The comparison between the estimated trajectory and the stage return value. (a) We used two ASI linear stages (Model: LS-50A) to translate the distal tip of the lensless fiber bundle to different positions. The trajectory (red dots) is estimated using the proposed cross-correlation analysis and the trajectory (blue dots) was provided through feedback from the motorized stages. (b) The error map for the estimated trajectory at different positions.

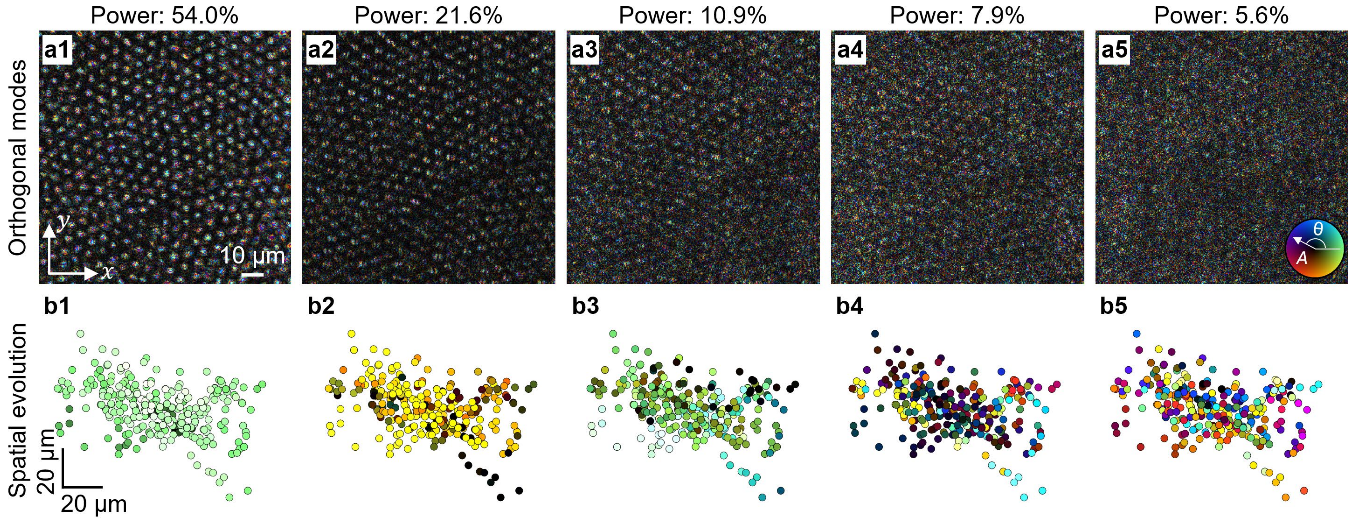

Fig. S17. Spatiotemporal decomposition of the modulation profile of the lensless fiber bundle. (a1-a5) The reconstructed spatiotemporal orthogonal modes $P_{st}(x, y)$, with $t = 1,2, \dots 5$. The amplitude is represented by greyscales, and color hues by phase information. The power is determined by $d_{st}/\sum_t d_{st}$ in percentage, where d_{st} is the tth diagonal element of D_s. (b1-b5) The evolution of v_{st} for each spatiotemporal orthogonal modes during the image acquisition process, where v_{st} presents the tth column of V_s.

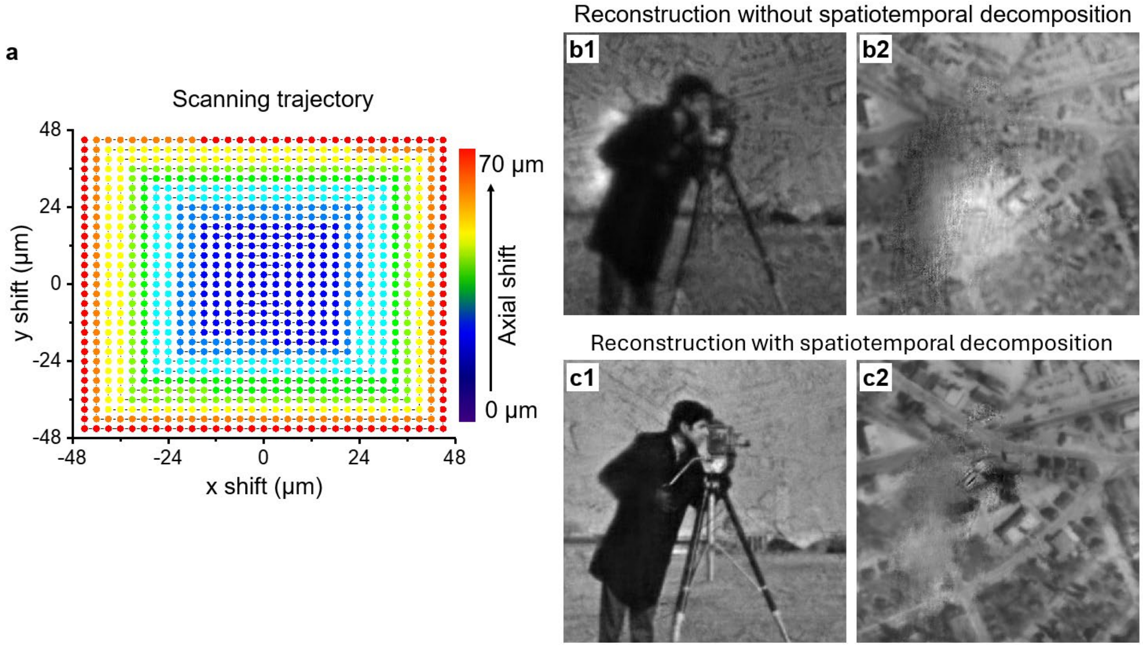

Fig. S18. A simulation study on axial movement compensation via spatiotemporal decomposition of the modulation profile. (a) The scanning trajectory of the object with different defocused distances for different lateral positions. In the simulation, the axial position changes gradually with each scan step, starting from an initial position of 0 and moving 0.08 microns with each step up to a maximum of 70 microns. (b) SAPE reconstruction without spatiotemporal decomposition, applying a consistent modulation profile for the fiber bundle at all translated positions. (c) SAPE reconstruction with spatiotemporal decomposition implemented. With the recovery of the orthogonal modes of the modulation profile, it may be possible to track the 3D trajectory of the endoscope probe. Future research along this line is highly desired.

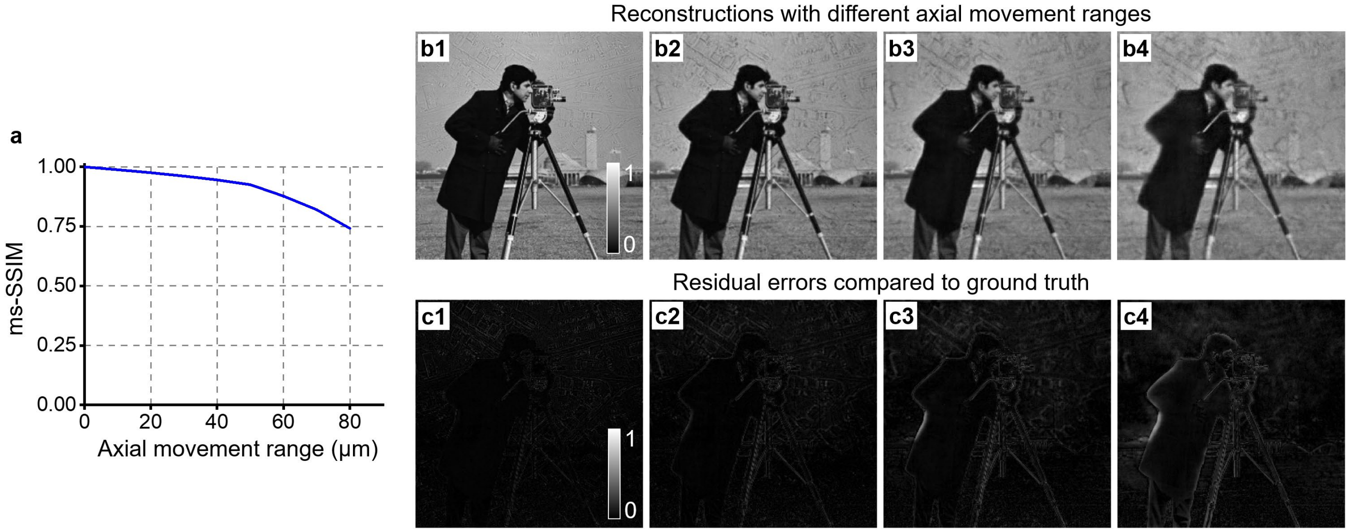

Fig. S19. Impact of axial movement on reconstruction quality. (a) Multiscale Structural Similarity Index (ms-SSIM) is used to quantify the performance of reconstructions compared to a reference reconstruction without axial movement (ground truth). (b1-b4) Reconstructions obtained using SAPE with various axial movement ranges: (b1) 10 µm, (b2) 30 µm, (b3) 50 µm, and (b4) 70 µm. (c1-c4) Residual error maps compared to the ground-truth reconstruction.

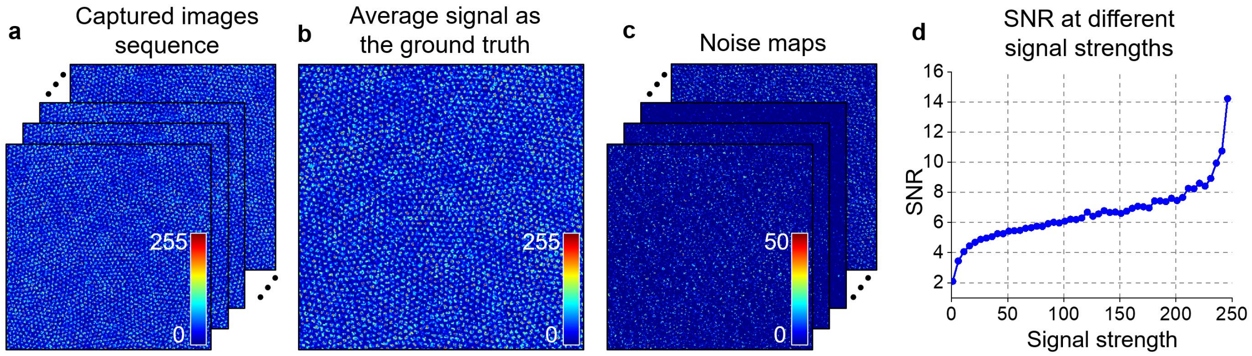

Fig. S20. Signal-to-noise ratio (SNR) analysis of SAPE with a stationary fiber bundle and a blood smear target. The SNR is calculated using the following steps: 1) Compute the mean image (μ) from 100 repeated measurements to represent the signal. 2) Calculate the difference between each individual image and the mean image to represent the noise (σ). 3) Determine the SNR as the ratio of the signal to the noise (SNR = μ / σ). (a) Examples of captured raw images with different signal strengths. (b) The corresponding mean images representing the signal (μ). (c) The noise images (σ) obtained by subtracting the mean image from each individual image. (d) The calculated SNR values at different input signal strengths, demonstrating the dependence of SNR on the signal strength in SAPE. The maximum achieved SNR is approximately 14 for the highest signal strength in our experiments.

References:

1. Wang T, Song P, Jiang S, Wang R, Yang L, Guo C*, et al.* Remote referencing strategy for high-resolution coded ptychographic imaging. *Optics Letters* 2023, **48**(2).
2. Zhang H, Jiang S, Liao J, Deng J, Liu J, Zhang Y*, et al.* Near-field Fourier ptychography: super-resolution phase retrieval via speckle illumination. *Optics express* 2019, **27**(5)**:** 7498-7512.
3. Faulkner HML, Rodenburg J. Movable aperture lensless transmission microscopy: a novel phase retrieval algorithm. *Physical review letters* 2004, **93**(2)**:** 023903.
4. Maiden A, Johnson D, Li P. Further improvements to the ptychographical iterative engine. *Optica* 2017, **4**(7)**:** 736-745.
5. Rodenburg J, Maiden A. Ptychography. *Springer Handbook of Microscopy*. Springer, 2019, pp 819-904.
6. Jiang S, Song P, Wang T, Yang L, Wang R, Guo C*, et al.* Spatial- and Fourier-domain ptychography for high-throughput bio-imaging. *Nature Protocols* 2023, **18**(7)**:** 2051-2083.
7. https://figshare.com/articles/figure/SAPE/25854430.
8. Stockmar M, Cloetens P, Zanette I, Enders B, Dierolf M, Pfeiffer F*, et al.* Near-field ptychography: phase retrieval for inline holography using a structured illumination. *Scientific reports* 2013, **3**(1)**:** 1-6.
9. Zheng G, Horstmeyer R, Yang C. Wide-field, high-resolution Fourier ptychographic microscopy. *Nature photonics* 2013, **7**(9)**:** 739.
10. Chang H, Enfedaque P, Marchesini S. Blind ptychographic phase retrieval via convergent alternating direction method of multipliers. *SIAM Journal on Imaging Sciences* 2019, **12**(1)**:** 153-185.
11. Fannjiang A, Chen P. Blind ptychography: uniqueness and ambiguities. *Inverse Problems* 2020, **36**(4)**:** 045005.
12. Jiang S, Zhu J, Song P, Guo C, Bian Z, Wang R*, et al.* Wide-field, high-resolution lensless on-chip microscopy via near-field blind ptychographic modulation. *Lab on a Chip* 2020, **20**(6)**:** 1058-1065.
13. Jiang S, Guo C, Song P, Wang T, Wang R, Zhang T*, et al.* High-throughput digital pathology via a handheld, multiplexed, and AI-powered ptychographic whole slide scanner. *Lab on a Chip* 2022, **22**(14)**:** 2657-2670.
14. Bian Z, Guo C, Jiang S, Zhu J, Wang R, Song P*, et al.* Autofocusing technologies for whole slide imaging and automated microscopy. *Journal of Biophotonics* 2020, **13**(12)**:** e202000227.
